# The Neuroimmune Axis in Gastric Cancer: Bridging Neural Regulation, Tumor Microenvironment, and Immunotherapy

**DOI:** 10.32604/or.2025.074893

**Published:** 2026-02-24

**Authors:** Fangyuan Zhang, Xi Wang, Xinxin Shen, Pei Xiong, Yan Yang, Jincheng Wang

**Affiliations:** 1 School of Pharmaceutical Science and Technology, Hangzhou Institute for Advanced Study, University of Chinese Academy of Sciences, Hangzhou, 310058, China; 2 The First Clinical Medical College, Nanjing University of Chinese Medicine, Nanjing, 210023, China; 3 School of Pharmacy, Shanghai University of Traditional Chinese Medicine, Shanghai, 201203, China; 4 Department of Health Promotion, China Science and Technology, Development Center of Chinese Medicine, Beijing, 100023, China; 5 Institute of Chinese Medicine Literature, Nanjing University of Chinese Medicine, Nanjing, 210023, China

**Keywords:** Gastric cancer, neuro-immune axis, tumor microenvironment, adrenergic signaling, immunotherapy resistance, microbiota-gut-brain axis

## Abstract

Accumulating evidence indicates that the neuro-immune axis is central to gastric cancer pathogenesis. Dynamic, bidirectional signaling between neural circuits and immune cells promotes tumor progression, shapes an immunosuppressive microenvironment, and contributes to therapeutic resistance. We synthesize current knowledge on how autonomic (sympathetic and parasympathetic) and sensory innervation regulate gastric cancer biology. These circuits act through neurotransmitters (catecholamines, acetylcholine) and neuropeptides (substance P [SP], calcitonin gene-related peptide [CGRP]) to foster tumor growth and angiogenesis, facilitate perineural invasion, and enable immune evasion by recruiting suppressive myeloid and lymphoid populations and by inducing checkpoint molecule expression. We also examine how chronic stress and the microbiota–gut–brain axis intensify immunosuppression via glucocorticoid signaling and microbially derived metabolites. In parallel, we discuss why current immunotherapies achieve only modest response rates (approximately 10%–20%) in many settings, emphasizing neurally mediated mechanisms of resistance. We evaluate therapeutic strategies that target the neuro-immune axis—including pharmacological neuromodulation, selective neural ablation, and rational combination regimens—and outline how single-cell approaches and neural-tumor-microenvironment organoid models can accelerate mechanism-driven translation. This review aims to integrate current evidence from neuroscience and immuno-oncology to construct a conceptual framework for neuro-immune regulation in gastric cancer and to identify potential therapeutic strategies to overcome treatment resistance by targeting neural–tumor–immune crosstalk.

## 1 Introduction

Gastric cancer remains a major global health challenge, characterized by high morbidity and mortality with limited therapeutic efficacy, particularly in advanced disease. Although surgical techniques, chemotherapy, and immunotherapy have advanced, outcomes for many patients remain poor, underscoring the need to define the biological mechanisms that drive tumor progression and immune escape [1]. Emerging research highlights a central role for the neuro-immune axis—a dynamic, bidirectional communication network between the nervous and immune systems—in shaping cancer biology. This axis integrates neural signaling with immune modulation and interactions within the tumor microenvironment, providing new insight into gastric cancer pathogenesis and revealing therapeutic vulnerabilities [2].

The stomach is densely innervated by autonomic and sensory neurons that regulate physiological functions such as motility, secretion, and blood flow. In malignancy, these circuits are co-opted to promote tumor growth, invasion, and immune suppression. The sympathetic nervous system—frequently engaged during chronic stress—drives gastric cancer progression via adrenergic pathways that enhance proliferation, angiogenesis, and metastatic dissemination. By contrast, parasympathetic inputs mediated by acetylcholine and vagal activity and show context-dependent effects: they can suppress inflammation in some settings yet facilitate tumor-permissive conditions in others [3]. Sensory neurons further contribute to the malignant milieu by releasing neuropeptides that influence pain perception, vascular remodeling, and immune cell behavior. Collectively, these neural inputs establish a niche that supports tumor survival and dissemination [4].

Beyond direct neural-tumor interactions, the neuro-immune axis strongly influences the tumor microenvironment (TME), where immune cells, stromal elements, and cancer cells engage in dense crosstalk. Neurotransmitters and neuropeptides modulate immune-cell programs and shift the balance between antitumor immunity and immunosuppression [5]. For example, sympathetic catecholamines skew macrophages toward pro-tumor phenotypes and blunt cytotoxic T-cell (CD8^+^ cytotoxic T lymphocyte, CD8^+^) activity [6]. Similarly, sensory neuron–driven neurogenic inflammation recruits myeloid-derived suppressor cells (MDSCs) and regulatory T cells (Tregs), further weakening immune surveillance [7]. These mechanisms may contribute to the limited efficacy of immune checkpoint inhibitors in gastric cancer and suggesting that targeting neuro-immune interactions could enhance therapeutic responses [8].

The gut-brain axis-mediated by the vagus nerve and influenced by the microbiome-adds an additional layer of complexity to neuro-immune regulation in gastric cancer [9]. The gut microbiota modulates neural signaling and immune responses, with potentially effects on tumor behavior and therapeutic outcomes. Dysbiosis—a feature of many gastrointestinal malignancies—can disrupt this balance and promote chronic inflammation and immune dysfunction [10]. In addition, chronic psychological stress activates neuroendocrine pathways and has been linked to accelerated tumor progression and reduced immunotherapy efficacy, underscoring the broader impact of neural and emotional states on cancer biology [11].

These insights have spurred interest in therapeutically targeting the neuro-immune axis in gastric cancer. Preclinical studies show that inhibiting adrenergic or cholinergic signaling reduces tumor growth and increases immune-cell infiltration, and that pharmacologic blockade of neuropeptide pathways can mitigate immunosuppression [12]. Neuromodulatory approaches—including vagus nerve stimulation and bioelectronic interventions—are being evaluated as adjuncts to conventional therapies [13,14]. Translational challenges remain. Key needs include precise target engagement to avoid disrupting essential neural functions and predictive biomarkers to guide patient selection. This review aims to integrate current evidence from neuroscience and immuno-oncology to construct a conceptual framework for neuro-immune regulation in gastric cancer and to identify potential therapeutic strategies to overcome treatment resistance by targeting neural–tumor–immune crosstalk.

## 2 Neural Regulation in Gastric Cancer

### 2.1 The Autonomic Nervous System in Gastric Cancer

The autonomic nervous system (ANS), comprising sympathetic and parasympathetic divisions, regulates gastric physiology and has emerged as a key modulator of tumor-intrinsic programs in gastric cancer. In this section, we focus on tumor-intrinsic consequences of autonomic signaling, particularly β-adrenergic pathways that promote proliferation, epithelial–mesenchymal transition, migration, and programmed death-ligand 1 (PD-L1) upregulation. Through direct neural-tumor interactions and systemic neuroendocrine signaling, the ANS influences multiple aspects of tumor biology, including cell proliferation, invasion, metastasis, and the tumor microenvironment [15,16]. The sympathetic nervous system, activated under conditions of chronic stress or inflammation, exerts predominantly tumor-promoting effects in gastric cancer [17]. Norepinephrine, the primary sympathetic neurotransmitter, activates β-adrenergic cascades in cancer cells, promoting proliferation, epithelial–mesenchymal transition, and metastatic dissemination via mitogen-activated protein kinase (MAPK) and cyclic AMP–protein kinase A–cAMP response element-binding protein (cAMP–PKA–CREB) signaling [18]. Operationally, β2-adrenergic input suppresses CD8^+^ and natural killer (NK) cell cytotoxicity, reduces dendritic-cell interleukin-12 (IL-12) and antigen presentation, skews CD4^+^ T cells toward Treg/Th2, and sustains MDSCs, while upregulating PD-L1 in tumor cells [19]. By contrast, parasympathetic inputs mediated by vagal activity and acetylcholine show context-dependent effects [20]. In myeloid cells, α7 nicotinic acetylcholine receptor (α7nAChR) engagement attenuates nuclear factor kappa-light-chain-enhancer of activated B cells (NF-κB) signaling and pro-inflammatory cytokines, whereas α7nAChR on tumor cells can activate phosphoinositide 3-kinase (PI3K)–protein kinase B (AKT)/extracellular signal–regulated kinase (ERK), supporting survival, migration, and perineural invasion [21]. Epidemiologic observations of reduced gastric-cancer incidence after vagotomy further suggest that parasympathetic tone can influence disease trajectories in specific contexts [22].

**Figure 1:**
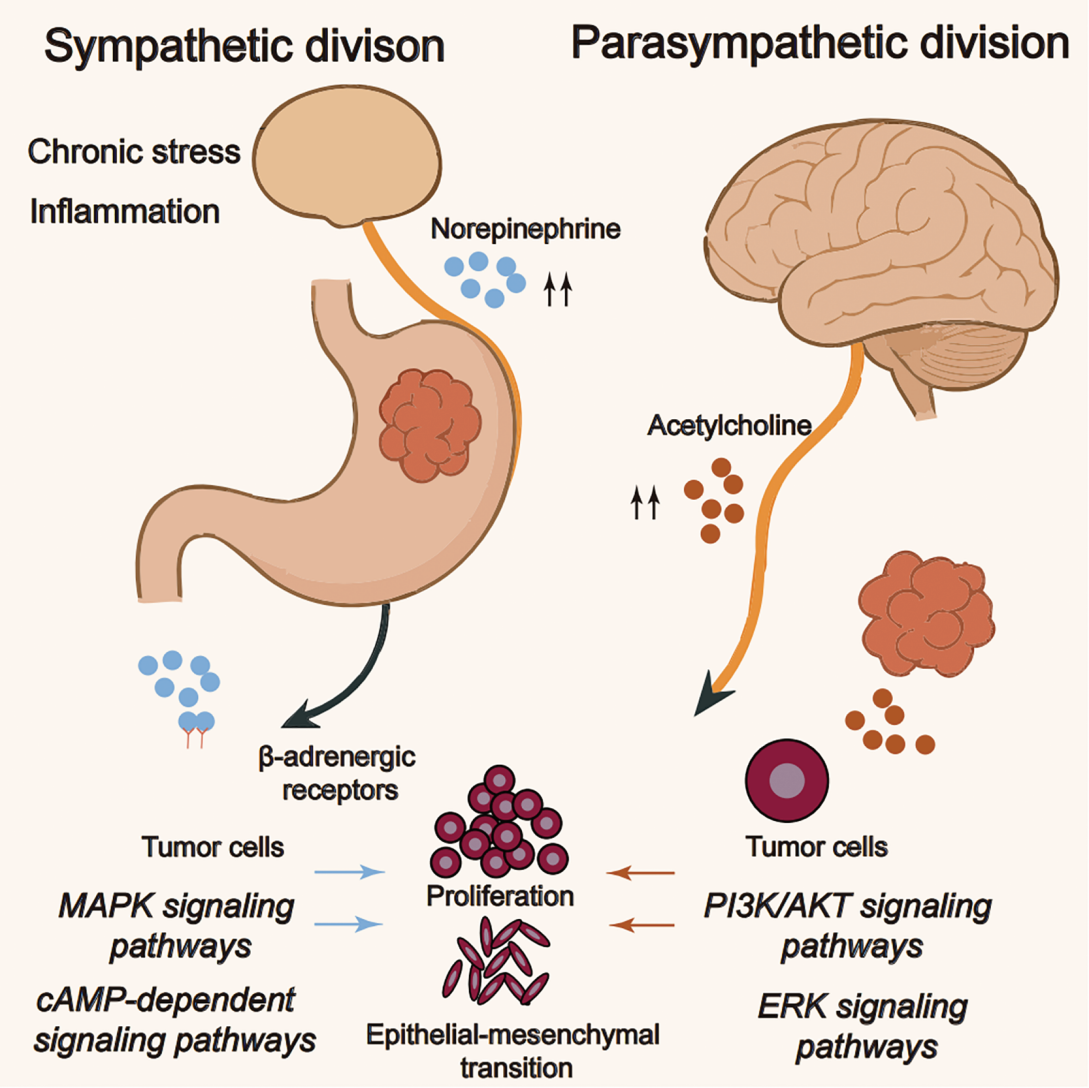
The autonomic nervous system in gastric cancer. Note: The schematic contrasts autonomic inputs to tumor cells. Under chronic stress or inflammation, sympathetic norepinephrine activates β-adrenergic receptors, engaging MAPK^1^ and cAMP–PKA–CREB^2^ pathways that promote proliferation and epithelial–mesenchymal transition. In parallel, parasympathetic acetylcholine activates PI3K/AKT^3^ and ERK^4^ signaling in tumor cells via nicotinic (α7nAChR^5^) and, where expressed, muscarinic receptors. The net influence of parasympathetic input is context-dependent, varying with disease stage and receptor distribution, whereas sympathetic signaling is generally tumor-promoting. This figure was created using Adobe Illustrator (version 27.9). ^1^MAPK: mitogen-activated protein kinase; ^2^cAMP: cyclic adenosine monophosphate; PKA: protein kinase A; CREB: cAMP response element-binding protein; ^3^PI3K: phosphoinositide 3-kinase; AKT: protein kinase B; ^4^ERK: extracellular signal-regulated kinase; ^5^α7nAChR: α7 nicotinic acetylcholine receptor

The balance between sympathetic and parasympathetic inputs dynamically shapes gastric cancer biology. In general, sympathetic dominance favors tumor progression, whereas parasympathetic activity exerts context-dependent effects that vary by stage and receptor distribution [23]. This neural regulation extends beyond direct effects on cancer cells to include modulation of the tumor microenvironment, angiogenesis, and immune responses. Neurotransmitters released by autonomic nerves can influence the behavior of stromal cells, endothelial cells, and immune cells within the tumor microenvironment, creating a permissive niche for tumor growth and dissemination [24]. Recognizing these neural influences is therefore essential for understanding gastric cancer pathogenesis and for rational therapy design [25]. Emerging evidence suggests that targeting autonomic signaling pathways, either through pharmacological modulation of neurotransmitter systems or through interventions affecting neural activity, may represent a promising approach for gastric cancer treatment, particularly in combination with conventional therapies or immunotherapies. Nonetheless, the precise mechanisms, optimal patient selection, and safety parameters require further investigation before these approaches can be translated reliably into clinical practice [26]. Fig. 1 schematically maps the autonomic and sensory inputs that shape gastric tumor ecology, including sympathetic β-adrenergic signaling, parasympathetic cholinergic/vagal pathways, and sensory-neuropeptide axes, with a stage-dependent sympathetic–parasympathetic balance.

### 2.2 Sensory Nerves and Pain Regulation in Gastric Cancer

#### 2.2.1 Sensory Innervation and Pain Mechanisms

Sensory-nerve involvement in gastric cancer is a critical yet understudied facet of tumor neurobiology, with dual roles in pain pathogenesis and modulation of the tumor microenvironment [27]. Sensory fibers arising from the dorsal root and trigeminal ganglia densely innervate the gastric mucosa and submucosa, and they undergo marked remodeling during carcinogenesis [28]. In gastric cancer, these fibers show altered distribution patterns, with increased density at the tumor periphery and selective depletion in necrotic tumor cores, suggesting dynamic interactions between growing malignancies and sensory innervation [29]. This spatial reorganization correlates with clinical pain manifestations, as tumor-associated neural sprouting and sensitization contribute to the characteristic visceral pain in advanced disease. The pain mechanisms involve both direct tumor compression of nerve bundles and molecular crosstalk through neuroactive substances released by cancer cells, such as ATP, protons, and growth factors, which activate sensory neurons through specific receptors (TRPV1, ASICs, and P2X3) [30].

#### 2.2.2 Substance P–NK1R Axis in Tumor Progression and Pain

SP and CGRP, the principal neuropeptides released by sensory nerves, serve as key mediators in the neuro-tumor dialogue. SP, acting via the neurokinin-1 receptor (NK1R), exerts multifaceted protumor effects in gastric cancer. NK1R-positive tumor cells respond to SP with increased proliferation and migration through mitogen-activated protein kinase/extracellular signal–regulated kinase (MAPK/ERK) pathway and NF-κB activation [31]. Within the vascular compartment, SP promotes angiogenesis by upregulating vascular endothelial growth factor (VEGF)in both cancer cells and tumor-associated macrophages and concurrently increases vascular permeability, thereby facilitating metastatic dissemination. SP also amplifies inflammation by inducing interleukin-6 (IL-6) and interleukin-8 (IL-8) secretion from stromal cells, generating a pro-tumorigenic cytokine milieu. Clinically, elevated SP levels in gastric cancer tissue correlate with advanced disease stage and poorer prognosis, suggesting its potential as both a therapeutic target and prognostic biomarker [32].

#### 2.2.3 CGRP Signaling and the Tumor Microenvironment

CGRP, a pivotal sensory neuropeptide, exerts context-dependent effects in gastric cancer. By binding to calcitonin receptor-like receptor/receptor activity-modifying protein (CLR/RAMP) complexes, CGRP modulates tumor-associated inflammation by skewing macrophage polarization towards an M2-like, pro-tumor phenotype. Its vasodilatory activity contributes to the aberrant vascular architecture typical of gastric tumors, and emerging data indicate direct mitogenic effects on cancer stem-like cells [33]. Paradoxically, CGRP may also exert anti-inflammatory effects in some contexts, highlighting the complexity of sensory neuropeptide signaling in cancer [34]. Both SP and CGRP promote pain via peripheral and central sensitization, lowering nociceptive thresholds and amplifying spinal pain transmission. The therapeutic implications of targeting sensory nerve-tumor interactions are increasingly evident. NK1R antagonists, originally developed as antiemetics, have shown preclinical efficacy in reducing gastric cancer growth and metastasis while alleviating cancer pain. Similarly, CGRP receptor antagonists, currently used for migraine treatment, may find application in managing gastric cancer-associated pain and potentially modifying tumor progression. Emerging approaches that combine neuromodulation with conventional treatments aim to simultaneously control tumor progression and cancer pain, offering a promising multidisciplinary strategy. Nevertheless, precise targeting of these pathways remains challenging, as interventions must avoid disrupting normal pain perception and sensory function [35].

Current research highlights the bidirectional nature of sensory nerve–tumor interactions: cancer cells not only respond to neuropeptides but also recruit and reprogram sensory nerves via nerve growth factor (NGF) and other neurotrophic factors, establishing a self-reinforcing cycle that drives tumor progression and pain chronification. Single-cell RNA sequencing studies are beginning to reveal the heterogeneity of sensory neuron subpopulations infiltrating gastric tumors, opening new possibilities for selective therapeutic intervention [36]. Furthermore, the intersection between sensory signaling and immune checkpoint regulation presents intriguing opportunities for combination therapies that might enhance the efficacy of existing immunotherapies while providing analgesia [37].

Defining the precise spatiotemporal dynamics of sensory-nerve involvement remains a central challenge. Advanced imaging techniques and neural tracing methods are being employed to map the anatomical and functional reorganization of sensory innervation during tumor progression [38]. These investigations may identify critical windows for therapeutic intervention and help develop biomarkers for predicting pain risk and progression patterns. As cancer neuroscience advances, targeting sensory nerve–tumor interactions could emerge as a fourth pillar of gastric-cancer therapy, complementing surgery, chemotherapy, and radiation [39] (Table 1).

**Table 1:** Neuropeptide-mediated interactions between sensory neurons and the tumor microenvironment

Category	Mechanisms & Effects	Clinical/Therapeutic implications	Reference
Sensory nerve distribution	Increased density at the tumor periphery	Correlates with pain intensity	[40]
	Depletion in necrotic cores	Potential imaging biomarker for tumor progression	[41]
	Remodeling via NGF^1^ signaling	N/A^2^	[42]
Pain mechanisms	Tumor compression	Visceral pain in 60%–80% advanced cases	[43]
	Neuroactive substances (ATP, protons)	Requires multimodal analgesia	[44]
SP^3^: Substance P	Binds NK1R^4^, activates MAPK/ERK^5^, NF-κB^6^	NK1R antagonists	[45]
	Promotes angiogenesis (VEGF^7^)	Prognostic biomarker	[46]
	Induces IL-6/IL-8^8^	Combined with anti-angiogenics	[47]
CGRP^9^: Calcitonin gene-related peptide	Binds CLR/RAMP^10^ receptors	CGRP antagonists (erenumab)	[48]
	M2 macrophage polarization	Potential anti-metastatic effects	[49]
Neuro-immune crosstalk	SP/CGRP modulate TAMs^11^	May enhance immunotherapy efficacy	[50]
	Interaction with PD-1/CTLA-4^12^ pathways	Risk of autoimmune side effects	[51]
Therapeutic strategies	NK1R/CGRP receptor blockers	Phase II trials for pain control	[52]
	Anti-NGF antibodies	Challenges in preserving protective nociception	[53]
Emerging research	Single-cell mapping of neuron subtypes	Personalized neuromodulation	[54]
	Neural tracing technologies	Combination with ICIs^13^	[55]
	Neuro-immune checkpoints	Bioelectronic medicine approaches	[56]

Note: ^1^NGF: nerve growth factor; ^2^N/A: Not applicable; ^3^SP: Substance P; ^4^NK1R: Neurokinin-1 receptor; ^5^MAPK/ERK: Mitogen-activated protein kinase/extracellular signal–regulated kinase; ^6^NF-κB: Nuclear factor kappa-light-chain-enhancer of activated B cells; ^7^VEGF: vascular endothelial growth factor; ^8^IL-6: Interleukin-6; IL-8: Interleukin-8; ^9^CGRP: Calcitonin gene-related peptide; ^10^CLR/RAMP: Calcitonin receptor-like receptor/receptor activity-modifying protein; ^11^TAMs: Tumor-associated macrophages; ^12^PD-1/CTLA-4: programmed cell death protein 1 (PD-1)/cytotoxic T-lymphocyte–associated protein 4 (CTLA-4). ^13^ICIs: Immune checkpoint inhibitors.

### 2.3 Innervation and Tumorigenesis in Gastric Cancer

Tumor innervation is a critical yet understudied dimension of gastric cancer progression, in which dynamic nerve–tumor interactions sculpt a tumor microenvironment conducive to growth, invasion, and metastasis. Emerging evidence suggests that neural infiltration into tumors is not merely a passive response to cancer expansion but an active process that contributes to disease pathogenesis. This bidirectional crosstalk involves specialized neuro-tumor interactions mediated by both direct synaptic communication and diffusible neurotrophic factors, which collectively shape the malignant phenotype of gastric cancer.

#### 2.3.1 Molecular Mechanisms of Neuro-Tumor Synapses

Recent advances in cancer neuroscience have revealed the existence of functional synaptic connections between neurons and cancer cells, termed “neuro-tumor synapses.” These specialized structures facilitate direct electrochemical communication through neurotransmitter release and receptor activation. In gastric cancer, such synapses predominantly involve glutamatergic and adrenergic signaling, where presynaptic neurons release neurotransmitters that bind to corresponding receptors on cancer cells. For instance, glutamate released from nerve terminals activates metabotropic glutamate receptors (mGluRs) on gastric cancer cells, triggering downstream MAPK and PI3K/AKT pathways that promote proliferation and resistance to apoptosis. Similarly, noradrenaline released from sympathetic nerve endings engages β-adrenergic receptors on tumor cells, activating cAMP/PKA signaling that enhances migratory capacity and metastatic potential [57].

The formation of neuro-tumor synapses is coordinated by adhesion molecules such as neuroligins and neurexins, which promote tight facilitate membrane apposition between nerve terminals and cancer cells. These molecular bridges are often overexpressed in innervated gastric tumors and correlate with perineural invasion—a histopathological feature associated with poor prognosis. Additionally, gap junctions composed of connexin proteins allow for direct ion and small molecule exchange between neurons and cancer cells, generating an electrically coupled network that synchronizes tumor cell behavior [58]. This synaptic mimicry enables cancer cells to co-opt neural circuits typically reserved for interneuronal communication, effectively turning nerves into conduits for protumor signaling.

Single-cell RNA sequencing studies have identified subsets of gastric cancer cells that express synaptic proteins typically restricted to neurons, including synaptophysin and postsynaptic density protein 95 (PSD-95). This “neuronal mimicry” suggests an evolutionary adaptation by which cancer cells optimize their ability to receive and process neural signals. Therapeutically, disrupting neuro-tumor synapses with targeted inhibitors of glutamatergic receptors or adrenergic signaling has shown promise in preclinical models, reducing tumor growth and neural invasion. However, selectively blocking these synapses without perturbing physiological neural function in healthy tissues remains a key challenge.

#### 2.3.2 Role of Neurotrophic Factors (Nerve Growth Factor [NGF], Brain-Derived Neurotrophic Factor [BDNF]) in Gastric Cancer Neural Infiltration

Neurotrophic Factors and Neural Infiltration in Gastric Cancer

Neurotrophic factors, particularly NGF and BDNF, serve as key mediators of neural infiltration in gastric cancer. Produced by tumor cells and stromal elements, these proteins establish chemotactic gradients that guide axonal growth toward and within the tumor.

NGF–TrkA Signaling in Neural Invasion and Tumor Progression

NGF, signaling through its high-affinity receptor tropomyosin receptor kinase A (TrkA)and the pan-neurotrophin receptor p75NTR, not only attracts sensory and sympathetic nerve fibers but also directly stimulates cancer cell proliferation and survival via PI3K/AKT and NF-κB activation. In gastric cancer, NGF expression correlates with the density of tumor-associated nerves and the degree of perineural invasion, highlighting its potential role as a biomarker of aggressive disease.

BDNF–TrkB Signaling and Dual Innervation

BDNF, acting through tropomyosin receptor kinase B (TrkB)receptors, exhibits complementary effects by promoting the outgrowth of enteric neurons that infiltrate gastric tumors. Unlike NGF, which primarily recruits extrinsic nerves, BDNF appears crucial for the expansion of intrinsic enteric nervous system fibers within the tumor microenvironment. This dual innervation—from both extrinsic and intrinsic sources—creates a dense neural network that supports tumor progression through multiple mechanisms. BDNF-TrkB signaling also protects cancer cells from anoikis (detachment-induced cell death), facilitating metastatic dissemination along nerve tracts [59].

Regulation of Neurotrophic Signaling and Therapeutic Targeting

The production of neurotrophic factors by gastric cancer cells is frequently stimulated by hypoxia and inflammatory cytokines, establishing a feed-forward loop in which tumor hypoxia induces NGF and BDNF secretion that, in turn, promotes nerve infiltration and the release of additional growth-promoting neurotransmitters. This self-reinforcing cycle underscores the therapeutic potential of targeting neurotrophic signaling. Monoclonal antibodies against NGF and small-molecule Trk inhibitors have demonstrated efficacy in preclinical gastric cancer models, reducing neural invasion and tumor progression. However, their clinical application requires careful consideration of potential neurological adverse effects, given the pleiotropic roles of neurotrophins in normal nervous system function.

Immunomodulatory Effects of Neurotrophic Factors

Neurotrophic factors also modulate the immune landscape in gastric tumors. NGF can polarize macrophages toward an immunosuppressive M2 phenotype, whereas BDNF has been shown to inhibit cytotoxic T-cell activity. These immunomodulatory effects underscore how neural infiltration can establish an immune-privileged niche that favors tumor progression. Current research is exploring combination therapies that simultaneously target neurotrophic signaling and immune checkpoints to disrupt both the neural and immunosuppressive components of the tumor microenvironment.

Clinical Implications and Future Directions

The clinical implications of tumor innervation are profound. Histopathological measures of neural density and perineural invasion status are increasingly recognized as independent prognostic factors in gastric cancer. Moreover, positron emission tomography (PET) radiotracers targeting neurotrophin receptors may soon enable noninvasive mapping of tumor innervation patterns, potentially for patient selection and treatment planning. As cancer neuroscience advances, therapies targeting the neural ecosystem of tumors may become integral to precision oncology approaches for gastric cancer. Future priorities include delineating the heterogeneity of tumor-associated nerves and engineering biomaterials for local delivery of neuromodulators to minimize systemic toxicity [60].

This evolving paradigm casts nerves as active components of the tumor microenvironment and opens therapeutic opportunities to disrupt reciprocal signaling between neurons and cancer cells. By targeting the molecular mechanisms of innervation, we may not only inhibit tumor progression but also prevent the debilitating pain associated with neural invasion in advanced gastric cancer (Table 2).

**Table 2:** Neural innervation mechanisms in gastric cancer tumorigenesis

Category	Key molecules/Pathways	Biological effects	Therapeutic implications	Reference
Neuro-tumor synapses				
Neurotransmitters	Glutamate (mGluRs), Noradrenaline (β-ARs)	Activates MAPK/PI3K pathways and proliferation	mGluR/β-AR inhibitors	[61]
		Enhances migration/metastasis	Synaptic adhesion blockers	
Adhesion molecules	Neuroligins, Neurexins	Facilitate membrane apposition between nerves and cancer cells	Antibody-based disruption of neuro-cancer junctions	[62]
Gap junctions	Connexins	Ion/small molecule exchange and synchronized tumor behavior	Connexin inhibitors (carbenoxolone)	[63]
Neurotrophic factors				
NGF/TrkA/p75NTR^1^	PI3K/AKT, NF-κB	Axonal guidance and neural infiltration	Anti-NGF antibodies (tanezumab)	[64]
		Anti-apoptosis and tumor survival	Trk inhibitors (larotrectinib)	[65]
BDNF^2^/TrkB	Enteric neuron expansion	Promotes perineural invasion	TrkB-targeted therapies	[66]
		Suppresses anoikis and metastasis		[67]
Hypoxia-inflammation link	HIF-1α, IL-6/TNF-α^3^	Upregulates NGF/BDNF secretion and feed-forward loop	Combined hypoxia/neurotrophin blockade	[68]
Clinical correlations				
Pathology markers	Perineural invasion (PNI), Synaptophysin/PSD-95	Predicts poor prognosis and recurrence	PNI as a histopathological biomarker	[69]
Imaging	PET tracers for Trk/neurotrophin receptors	Non-invasive mapping of tumor innervation	Guided treatment stratification	[70]
Combination strategies	PD-1/CTLA-4 inhibitors and Neuro-modulators	Overcomes neural-mediated immunosuppression	Phase I/II trials targeting neuro-immune crosstalk	[71]

Note: ^1^NGF: Nerve growth factor; TrkA: tropomyosin receptor kinase A; p75NTR: p75 neurotrophin receptor; ^2^BDNF: Brain-derived neurotrophic factor; ^3^HIF-1α: Hypoxia-inducible factor-1 alpha; TNF-α: Tumor necrosis factor-alpha.

## 3 Neuroimmune Axis and Tumor Microenvironment

### 3.1 Neuro-Immune Cell Interactions in Gastric Cancer

In this section, we examine the immune system consequences of neural inputs. Bidirectional crosstalk between neural circuits and immune cells constitutes a critical axis in the gastric-cancer microenvironment, dynamically shaping antitumor immunity and immunosuppressive networks. This neuro-immune dialogue occurs through multiple molecular channels, with neural-derived signals directly modulating immune cell function and phenotype, while immune cells reciprocally influence neuronal activity through cytokine release. The resulting network complexity materially contributes to immune evasion during gastric cancer progression.

#### 3.1.1 Neural Regulation of Immune Cells

Neural signaling exerts profound and cell-type-specific effects on tumor-infiltrating immune populations through both neurotransmitter-mediated pathways and neuropeptide-receptor interactions. Macrophages, as key orchestrators of the tumor microenvironment, demonstrate remarkable plasticity in response to neural inputs. Catecholamines from sympathetic nerves polarize macrophages toward an M2-like phenotype via β2-adrenergic receptor (β2-AR) signaling in preclinical models, resulting in increased production of immunosuppressive cytokines (interleukin-10 [IL-10], transforming growth factor–β, [TGF-β]) and decreased antigen presentation capacity [72]. This adrenergic skewing is further enhanced by neuropeptide Y (NPY) *in vitro* and *in vivo*, which synergizes with catecholamines to upregulate arginase-1 expression, promoting tumor-associated macrophage (TAM) differentiation. Conversely, parasympathetic acetylcholine signaling throughα7nAChR has been shown in organoid/*ex vivo* and murine systems to attenuate M1 response, often tilting established tumors toward tolerance [73].

T cell populations are highly susceptible to neural modulation. Sympathetic overactivation induces CD8^+^ T cell exhaustion through cAMP-PKA pathway activation, characterized by increased expression of inhibitory receptors (programmed cell death protein 1 [PD-1], T cell immunoglobulin and mucin-domain containing-3 [TIM-3]) and impaired cytotoxic function. By contrast, β-adrenergic (β-AR) signaling expands and activates regulatory T cells (Tregs), enhancing their suppressive capacity through FoxP3 upregulation. The neurotransmitter dopamine presents an intriguing exception, exhibiting dual context-dependent effects—while low doses suppress T cell activation, physiological concentrations may actually enhance CD8^+^ T cell memory formation, suggesting a potential therapeutic opportunity for optimized dosing strategies [74].

MDSCs are particularly sensitive neural targets in gastric cancer. SP signaling through NK1R not only recruits MDSCs to tumors but also augments their suppressive activity by upregulating arginase-1 and inducible nitric oxide synthase (iNOS). This SP-MDSC axis establishes a feed-forward loop, as MDSC-derived IL-6 further stimulates neuronal outgrowth and neuropeptide release. The resulting neural-immune circuit represents a formidable barrier to effective anti-tumor immunity and may contribute to the limited efficacy of current immunotherapies in gastric cancer.

#### 3.1.2 Neural Control of Immune Checkpoint Expression

Beyond direct immune cell modulation, neural signals significantly influence the expression of immune checkpoint molecules that govern tumor-immune interactions. Norepinephrine upregulates PD-L1 on gastric cancer cells and antigen-presenting cells in preclinical models via β-AR–cAMP–CREB signaling, contributing to a ‘cold’ TME. This adrenergic-PD-L1 axis, supported by preclinical evidence, operates independently of interferon-γ signaling, representing an alternative mechanism of immune checkpoint regulation that may require combined β-blockade and PD-1/PD-L1 inhibition for optimal disruption.

Neuropeptides also reshape the immune-checkpoint landscape. Vasoactive intestinal peptide (VIP) induces PD-L1 and CTLA-4 on dendritic cells while simultaneously downregulating co-stimulatory molecules (CD80, CD86), effectively impairing T-cell priming. In gastric cancer specimens, VIP-positive nerve fibers show striking spatial colocalization with PD-L1+ tumor regions, suggesting local neural control of immune evasion. Similarly, CGRP upregulates multiple checkpoints (PD-L1, LAG-3) on myeloid cells through CLR/RAMP receptor signaling, creating a multi-inhibitory environment.

The clinical implications of these findings are substantial. Preclinical models indicate that pharmacological β-blockademay enhance the activity of anti-PD-1 therapy in gastric cancer, while antagonism of the SP receptor reduces MDSC accumulation and checkpoint expression. Emerging evidence suggests that neural regulation of immune checkpoints can contribute to the development of immunotherapy resistance, as denervation experiments show restored sensitivity to checkpoint blockade in previously refractory tumors. Current clinical trials are exploring these neuro-immune interactions with combination regimens that target both neural signaling and immune checkpoints, and early reports describe improved response rates relative to monotherapy.

Technological advances are rapidly deepening our understanding of neuro-immune crosstalk. Spatial transcriptomics delineates anatomical relationships between nerve fibers and immune-cell clusters within gastric tumors, while optogenetic tools allow precise manipulation of neural activity to study real-time immune consequences. Together, these approaches reveal marked heterogeneity in neuro-immune interactions across gastric-cancer subtypes, suggesting future therapeutic strategies may require personalized neuromodulation tailored to tumor innervation patterns and immune profiles [75]. As the field progresses, targeting the neuro-immune axis may provide a crucial avenue for overcoming the current limitations of gastric cancer immunotherapy (Table 3).

**Table 3:** Neuro-immune interactions in gastric cancer microenvironment

Target cell/Process	Neural signal	Receptor/Pathway	Immunomodulatory effects	Therapeutic opportunities	Reference
**Macrophages**					
M2 polarization	Norepinephrine	β2-AR/cAMP^1^	↑IL-10, TGF-β; ↓Antigen presentation	β-blockers (propranolol) + CSF1R inhibitors	[76]
Metabolic reprogramming	Neuropeptide Y	NPY-R1^2^	↑Arginase-1 activity	NPY receptor antagonists^3^	[77]
**T Cells**					
CD8^+^ Exhaustion	Norepinephrine	β-AR/PKA^4^	↑PD-1, TIM-3^5^; ↓Cytotoxicity	β-blockers + anti-PD-1	[78]
Treg activation	Norepinephrine	β2-AR^6^	↑FoxP3, IL-35 secretion^7^	Selective β2-antagonists	[79]
Memory formation	Dopamine	DRD1/DRD2^8^	Context-dependent modulation	Low-dose dopamine agonists	[80]
**MDSCs^9^**					
Recruitment/Activation	SP	NK1R^10^	↑Arginase-1, iNOS^11^	Aprepitant (NK1R antagonist)	[81]
Immunosuppression	CGRP	CLR/RAMP	↑PD-L1, ROS production	CGRP receptor blockers	[82]
**Immune checkpoints**					
PD-L1 upregulation	Norepinephrine	β-AR/CREB	IFN-γ-independent induction^12^	β-blocker + immunotherapy combo	[83]
Dendritic cell anergy	VIP	VPAC1/2^13^	↑PD-L1/CTLA-4; ↓CD80/86	VIPhyb (VIP antagonist)	[84]
Myeloid checkpoints	CGRP	RAMP1/CLR	Co-upregulation of PD-L1/LAG-3^14^	Anti-CGRP mAbs (erenumab)	[85]
**Clinical correlations**					
Spatial organization	Nerve fibers	–	Correlation with “cold” tumors	Nerve density as biomarker	[86]
Therapy resistance	Neural activity	–	Primary/adaptive immunotherapy resistance	Denervation + ICB trials	[87]

Note: ^1^β2-AR/cAMP: β2-adrenergic receptor (β2-AR)/cyclic adenosine monophosphate (cAMP) (β2-AR/cAMP); ^2^NPY-R1: neuropeptide Y receptor 1; ^3^CSF1R: Colony-stimulating factor 1 receptor; NPY: Neuropeptide Y; ^4^β-AR: β-adrenergic receptor; PKA: Protein kinase A; ^5^TIM3: T cell immunoglobulin and mucin-domain containing-3; ^6^β2-AR: β2-adrenergic receptor; ^7^FoxP3: Forkhead box P3; IL-35: interleukin-35; ^8^DRD1: dopamine receptor D1; DRD2: dopamine receptor D2; ^9^DSCs: myeloid-derived suppressor cells; ^10^NK1R: neurokinin-1 receptor; ^11^iNOS: inducible nitric oxide synthase; ^12^IFN-γ: Interferon-γ. ^13^VPAC1: vasoactive intestinal peptide receptor 1; VPAC2: vasoactive intestinal peptide receptor 2; ^14^LAG-3: lymphocyte activation gene 3.

### 3.2 Chronic Stress and Immunosuppression in Gastric Cancer

The growing recognition of brain-tumor interactions highlights chronic stress as a significant modulator of gastric-cancer progression through its effects on the tumor immune microenvironment. This neuroendocrine-immune axis operates through two interconnected yet distinct mechanisms: hormonal regulation via stress-activated pathways and neuroinflammatory remodeling of the tumor niche.

#### 3.2.1 Stress Hormones and TME Immunosuppression

Chronic stress activates the hypothalamic-pituitary-adrenal (HPA) axis and sympathetic nervous system, driving sustained secretion of glucocorticoids (primarily cortisol) and catecholamines (norepinephrine and epinephrine). These stress hormones remodel the gastric tumor microenvironment through multiple, parallel mechanisms. Cortisol exerts immunosuppressive effects predominantly via the glucocorticoid receptor, inducing transcriptional programs in immune cells that favor tumor immune evasion. MDSCs show particular sensitivity to glucocorticoid signaling, with cortisol promoting their expansion and enhancing their immunosuppressive capacity through upregulation of arginase-1 and inducible nitric oxide synthase. The differentiation and function of dendritic cells are similarly impaired, resulting in deficient antigen presentation and reduced T cell priming. Natural killer cells, critical mediators of tumor immune surveillance, exhibit decreased cytotoxic activity and interferon-γ production under chronic glucocorticoid exposure. Perhaps most significantly, the T cell compartment undergoes dramatic reprogramming, with cortisol promoting regulatory T cell (Treg) expansion while inhibiting Th1 differentiation and CD8^+^ T cell effector function. Collectively, this hormonal modulation establishes an immune-tolerant microenvironment that permits tumor progression. Complementing these effects, catecholamines exert acute immunosuppression actions through β-adrenergic receptor signaling. Norepinephrine and epinephrine activate the cAMP-PKA pathway in macrophages, biasing polarization toward an M2-like phenotype characterized by increased IL-10 and TGF-β secretion. In T lymphocytes, chronic adrenergic signaling promotes an exhaustion phenotype with co-expression of multiple inhibitory receptors, including PD-1, TIM-3, and LAG-3. The discovery that β-adrenergic signaling can directly upregulate PD-L1 on both tumor cells and myeloid cells through CREB-dependent transcription provides a mechanistic link between stress and immunotherapy resistance. Clinical observations support these experimental findings, with elevated stress hormone levels correlating with poorer responses to immune checkpoint inhibitors in gastric cancer patients.

#### 3.2.2 Neuroinflammation and Immune Escape

Beyond endocrine mechanisms, chronic stress induces persistent neuroinflammation that further facilitates immune evasion in gastric cancer. This neuroinflammatory cascade begins with microglial activation in stress-responsive brain regions and is accompanied by increased production of proinflammatory cytokines, including IL-1β, TNF-α, and IL-6. These cytokines enter systemic circulation and infiltrate the tumor microenvironment, where they activate NF-κB and STAT3 signaling pathways in both tumor and stromal cells. The resulting inflammatory milieu promotes recruitment and activation of immunosuppressive cell populations while inhibiting effective anti-tumor immunity [88].

Sensory neurons within and around gastric tumors amplify inflammatory signaling by releasing neuropeptides such as SP and CGRP. SP, acting through neurokinin-1 receptors, induces pro-tumorigenic cytokines and chemokines while directly inhibiting T cell activation. CGRP exerts complementary effects by promoting the differentiation and recruitment of regulatory T cells and tolerogenic dendritic cells. Together, these neuropeptides sustain a self-reinforcing neurogenic inflammation loop that maintains an immune-permissive milieu [75].

The gut-brain axis further modulates neuroinflammatory through bidirectional communication between the enteric nervous system and central stress circuits. Stress-induced alterations in gut permeability and shifts in microbiota composition heighten systemic exposure to microbial products, thereby amplifying peripheral inflammation and neuroimmune activation. In gastric cancer, these changes manifest as elevated production of damage-associated molecular patterns and activation of pattern recognition receptors that can promote tumor progression.

Emerging therapeutic strategies aim to interrupt these stress-induced immunosuppressive pathways. β-adrenergic blockade has shown promise in preclinical models by restoring immune function and enhancing checkpoint inhibitor efficacy. Similarly, pharmacological modulation of neuropeptide signaling and targeted anti-inflammatory approaches may help break the cycle of neurogenic immunosuppression. Development of biomarkers to identify patients with stress-related immune dysfunction could enable more personalized treatment in gastric cancer [89].

### 3.3 The Microbiota-Gut-Brain Axis in Gastric Cancer Pathogenesis

#### 3.3.1 Microbiota–Gut–Brain Communication and Neural Pathways

Emerging insights into the microbiota-gut-brain axis implicate it in gastric cancer development through complex, bidirectional communication among intestinal microbes, the enteric nervous system, and central neural circuits. This trilateral network influences gastric tumor immunology through neural and humoral pathways, helping establish a microenvironment that shapes tumor progression and therapeutic responsiveness. The vagus nerve is the principal anatomical conduit for gut-brain communication, with afferent fibers transmitting microbial signals from the gastrointestinal tract to the central nervous system and efferent fibers carrying regulatory inputs back to the gut-associated lymphoid tissue [90]. Specific gut microbiota compositions can modulate gastric cancer immunity through vagus nerve-dependent mechanisms, where microbial metabolites such as short-chain fatty acids (SCFAs) and secondary bile acids activate enteric neurons that subsequently influence tumor-infiltrating immune cells. Butyrate-producing bacteria, for instance, stimulate vagal afferents that trigger anti-inflammatory responses in the gastric tumor microenvironment, while certain pathogenic species activate sympathetic outflow that suppresses cytotoxic T cell activity. This microbial-neural-immune crosstalk forms a dynamic regulatory system in which dysbiosis may promote tumor immune evasion through functional rewiring of neural circuits [91].

#### 3.3.2 Helicobacter pylori–Driven Neuro-Immune Interactions

*Helicobacter pylori* infection provides a particularly compelling example of neuro-immune interactions in gastric carcinogenesis. Beyond its established role in driving chronic inflammation and epithelial damage, *H. pylori* engages in sophisticated dialogue with both the enteric and central nervous systems. The bacterium produces virulence factors such as vacuolating cytotoxin A (VacA) and cytotoxin-associated gene A (CagA) that directly interact with enteric neurons, altering their excitability and neurotransmitter release profiles. These neural modifications subsequently influence the gastric immune landscape, with *H. pylori*-infected neurons releasing SP and calcitonin gene-related peptide that recruit immunosuppressive myeloid cells while inhibiting protective T cell responses [92]. Infection also induces structural plasticity of gastric innervation, increasing the density of sensory fibers that can sustain inflammatory signaling even after bacterial eradication. Notably, *H. pylori* can modulate central stress response pathways through vagus-mediated signaling, creating a feedback loop where psychological stress exacerbates infection-related carcinogenesis and *vice versa*. This neuro-immune interplay may help explain the variable outcomes of *H. pylori* infection, where some individuals develop protective immunological tolerance while others progress to premalignant lesions and eventually gastric adenocarcinoma [93].

#### 3.3.3 Microbial Metabolites, Neural Modulation, and Immune Regulation

The gut microbiome further shapes gastric-cancer progression through its metabolic output, with various microbial metabolites directly affecting neural function and immune regulation. Tryptophan metabolites along the kynurenine pathway, for example, act as mediators of this cross-talk by engaging aryl-hydrocarbon receptors on neural and immune cells and by modulating immune checkpoints to foster an immunosuppressive niche [93]. Gamma-aminobutyric acid (GABA) produced by gut bacteria can similarly modulate tumor-associated macrophages through GABA receptor signaling while simultaneously influencing central neural circuits involved in stress response. Collectively, these position the microbiota-gut-brain axis as an integrative regulator of gastric cancer immunology, in which microbial, neural, and immune factors coalesce to determine tumor fate.

#### 3.3.4 Therapeutic Implications and Holistic Management

**Figure 2:**
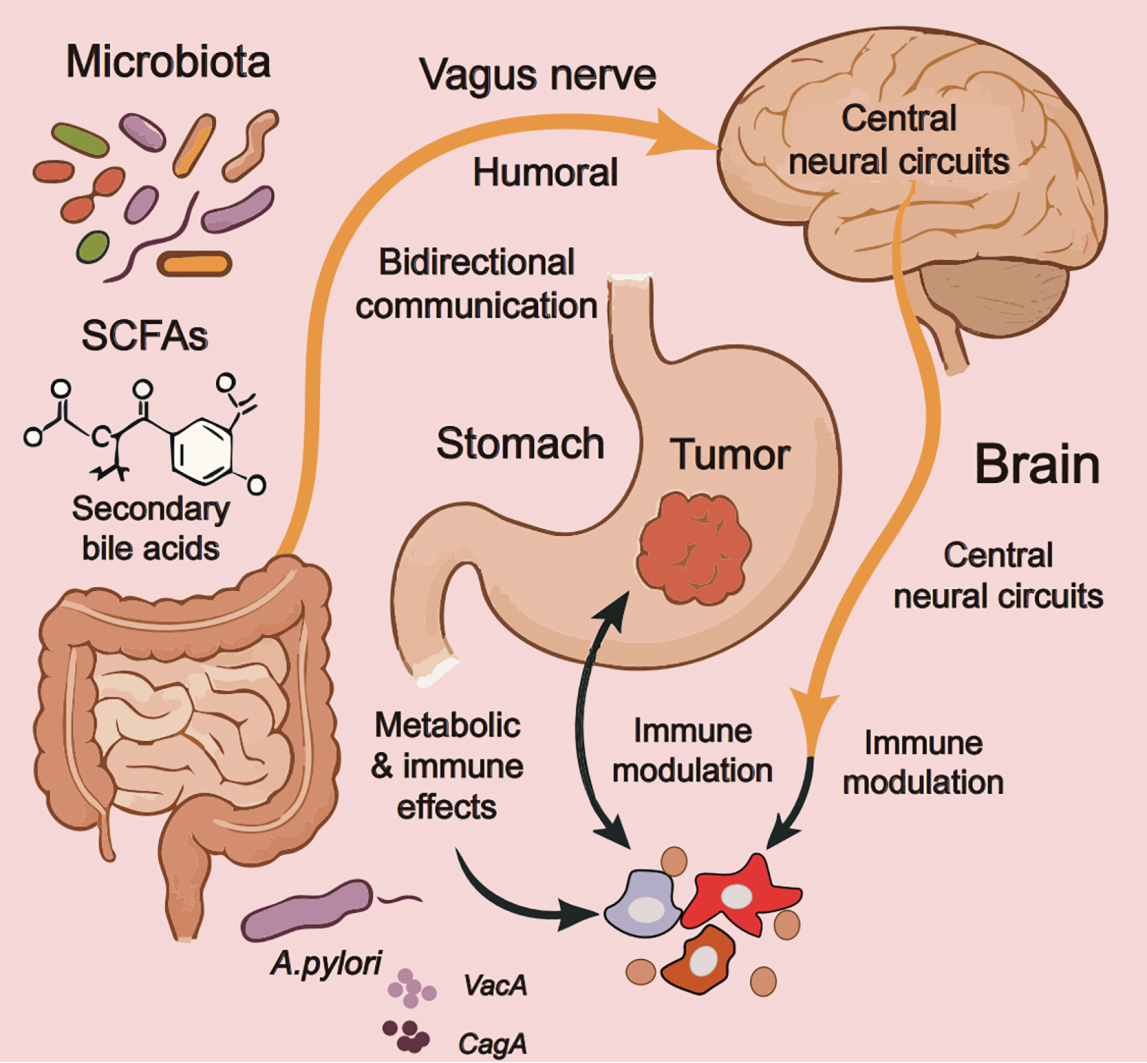
The microbiota-gut-brain axis in gastric cancer pathogenesis. Note: this schematic illustrates the complex bidirectional communication within the microbiota-gut-brain axis and its impact on gastric cancer pathogenesis. The diagram highlights key components, including gut microbiota metabolites (short-chain fatty acids [SCFAs]^1^, secondary bile acids), vagus nerve-mediated neural signaling, central neural circuit modulation, and specific H. pylori virulence factors (VacA^2^, CagA^3^) that collectively influence tumor immunology through integrated humoral and neural pathways. The figure demonstrates how microbial-neural-immune interactions create a dynamic regulatory network where dysbiosis can promote tumor progression via altered immune modulation and metabolic effects, while also emphasizing H. pylori’s unique role in disrupting neuroimmune homeostasis through direct neural interactions and systemic inflammatory responses. This figure was created using Adobe Illustrator (version 27.9). ^1^SCFAs: Short-chain fatty acids; ^2^VacA: Vacuolating cytotoxin A; ^3^CagA: Cytotoxin-associated gene A

Therapeutic manipulation of this axis through probiotics, vagus nerve stimulation, or microbial metabolite supplementation represents a promising frontier in gastric cancer treatment, potentially offering ways to overcome current limitations in immunotherapy efficacy [94]. Recognition of these complex interactions underscores the need for a more holistic approach to gastric-cancer management that considers not just the tumor itself but its broader neuroimmune and microbial context [95]. Fig. 2 illustrates the complex bidirectional communication within the microbiota–gut–brain axis and its impact on gastric cancer pathogenesis.

## 4 Targeting the Neuroimmune Axis for Immunotherapy

### 4.1 Limitations of Existing Immunotherapeutic Approaches

The clinical use of immune-checkpoint inhibitors in gastric cancer remains limited, reflecting the biological complexity of this malignancy. Despite representing a significant advancement in cancer treatment, PD-1/PD-L1 inhibitors demonstrate notably constrained efficacy in gastric cancer populations, with objective response rates typically remaining between 10%–20% in unselected patient cohorts. This modest performance stems from multiple interrelated factors that characterize the unique immunological landscape of gastric malignancies.

#### 4.1.1 Suboptimal Response Rates to PD-1/PD-L1 Inhibition

Heterogeneous PD-L1 expression across gastric cancer subtypes contributes to substantial variability in treatment responses. While the combined positive score (CPS) serves as a companion diagnostic, its predictive value is imperfect, and marked response heterogeneity persists even among CPS-positive patients. Diffuse-type carcinomas consistently fare worse than intestinal-type tumors after checkpoint inhibition, likely reflecting distinct microenvironment composition and lower immunogenicity. Tumor mutational burden, while predictive in some cancer types, demonstrates inconsistent correlation with immune therapy benefit in gastric cancer, partially because the specific mutagenic processes underlying different etiological subtypes (EBV-positive vs microsatellite unstable tumors) create divergent immunological contexts. The characteristic immunosuppressive tumor microenvironment of gastric cancer, rich in regulatory T cells, myeloid-derived suppressor cells, and M2-polarized macrophages, establishes multiple layers of immune resistance that single-agent checkpoint inhibition frequently fails to overcome [96].

#### 4.1.2 Neural Mechanisms of Immunotherapy Resistance

Emerging evidence identifies the nervous system as a critical mediator of immunotherapy resistance through multifaceted biological mechanisms. Neuron-derived signals actively remodel the tumor immune microenvironment in ways that counteract the intended effects of immune checkpoint blockade. Catecholamines released from sympathetic nerve terminals engage β-adrenergic receptors on immune and tumor cells, activating parallel immunosuppressive pathways that undermine PD-1/PD-L1 inhibition [97]. This adrenergic signaling induces compensatory expression of alternative immune checkpoints, including LAG-3 and TIM-3 on exhausted T cells while simultaneously expanding immunosuppressive myeloid populations [98].

Neuropeptide signaling imposes additional barriers to effective immunotherapy. SP and vasoactive intestinal peptide modulate dendritic cell function and promote regulatory T cell activity through distinct receptor-mediated pathways. The vagus nerve exerts immunosuppressive effects in the gastric microenvironment through cholinergic signaling that suppresses pro-inflammatory cytokine production and limits T cell activation. Preclinical models demonstrate that PD-1 blockade triggers compensatory increases in neural outgrowth and neurotransmitter release, suggesting an active neural response to immune activation [99].

The gut-brain axis contributes significantly to immunotherapy resistance through microbial-neural-immune interactions that are particularly relevant in gastric cancer. Specific gut microbiota compositions associated with poor responses appear to promote sympathetic tone and neuroinflammatory states that reinforce immune tolerance. Persistent *Helicobacter pylori* infection maintains chronic, low-grade inflammation and neural remodeling, thereby fostering an immunotherapy-resistant microenvironment. The enteric nervous system mediates this process by transmitting microbial signals to central neural circuits that subsequently modulate systemic immune responses through neuroendocrine pathways.

These complex neuro-immune interactions help explain both the modest initial response rates and the frequent loss of response to current immunotherapies. They imply that successful treatment strategies may require simultaneous targeting of both immune checkpoints and neural signaling pathways. Ongoing clinical trials investigating combinations of PD-1/PD-L1 inhibitors with β-adrenergic blockers, neurokinin receptor antagonists, and vagus nerve modulators may provide critical proof-of-concept for this integrated therapeutic approach. Developing reliable biomarkers to identify patients with neural-mediated resistance mechanisms represents another crucial direction for future research in this field [100].

### 4.2 Potential Therapeutic Targets and Intervention Strategies

#### 4.2.1 Targeting Neurotransmitter Receptors for Combination Therapy

The growing understanding of neural regulation in gastric cancer has identified several neurotransmitter receptors as promising therapeutic targets. β-adrenergic receptor blockade has emerged as a particularly compelling strategy, with preclinical studies demonstrating that drugs like propranolol can reverse catecholamine-mediated immunosuppression and enhance PD-1 inhibitor efficacy. Proposed mechanisms include reduced MDSC infiltration, decreased regulatory T-cell (Treg) activity, and normalization of aberrant tumor vasculature [101]. Clinical trials are currently investigating β-blockers in combination with immune checkpoint inhibitors for gastric cancer. Similarly, cholinergic antagonists targeting α7 nicotinic acetylcholine receptors (α7nAChR) show potential to mitigate the immunosuppressive effects of vagus nerve signaling [102]. These drugs appear to restore dendritic cell function and improve CD8^+^ T cell infiltration in gastric cancer models. The timing and dosing of these neuromodulatory agents relative to immunotherapy appear crucial, with some studies suggesting pretreatment with neural-targeting drugs may be necessary for optimal immune activation [103].

#### 4.2.2 Neuropeptide Inhibitors in Preclinical Development

SP/NK-1R signaling is increasingly recognized as a key pathway in gastric cancer progression and immune evasion. NK-1 receptor antagonists like aprepitant, originally developed as antiemetics, have demonstrated multifaceted antitumor effects in preclinical models [104]. These drugs not only curb cancer cell proliferation and migration but also significantly alter the immune landscape by decreasing MDSC accumulation and improving cytotoxic T cell function. In gastric cancer models, NK-1R blockade has been shown to reduce PD-L1 expression on tumor cells while increasing interferon-γ production by tumor-infiltrating lymphocytes. Other neuropeptide systems under investigation include CGRP receptor antagonists, which have shown potential to normalize tumor vasculature and reduce macrophage-mediated immunosuppression. Interestingly, some neuropeptide inhibitors appear to have direct effects on cancer stem-like cells, suggesting they may help prevent recurrence [105]. Optimal combinations with conventional therapies—and their impact on chemotherapy-induced neuropathy—remain active areas of investigation [106].

#### 4.2.3 Neural Ablation Techniques and Microenvironment Modulation

**Figure 3:**
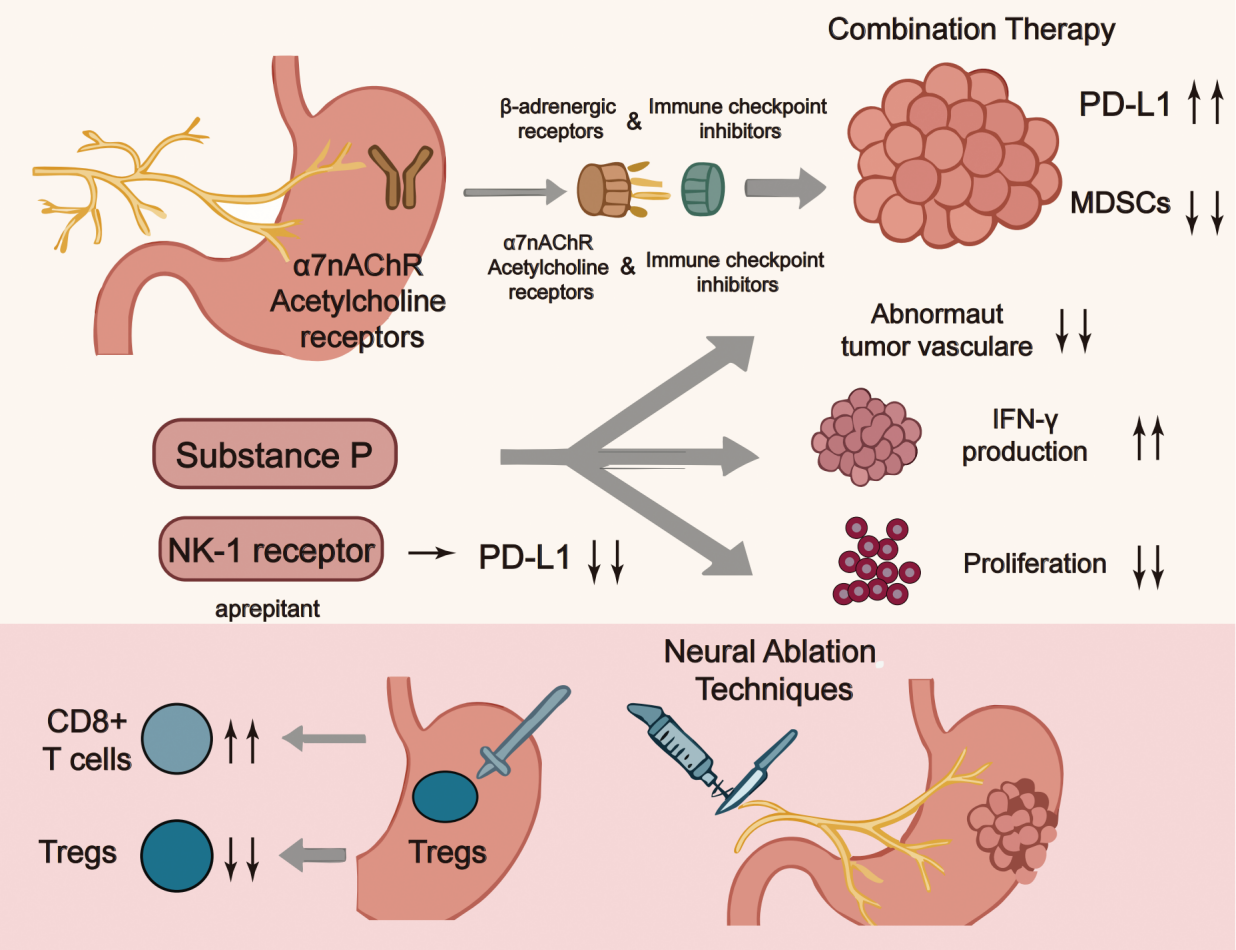
Targeting the neuroimmune axis in gastric cancer immunotherapy. Note: This schematic illustrates potential therapeutic strategies targeting the neuroimmune axis to enhance immunotherapy efficacy in gastric cancer. Key approaches include: (1) combination therapies pairing β-adrenergic receptor blockers or α7 nicotinic acetylcholine receptor (α7nAChR) antagonists with immune checkpoint inhibitors to counteract neural-mediated immunosuppression; (2) modulation of the tumor microenvironment by targeting PD-L1, myeloid-derived suppressor cells (MDSCs), and abnormal vasculature to improve T cell function; (3) inhibition of neuropeptide signaling, such as blocking the SP/NK-1 receptor axis with aprepitant, to reduce immune evasion and boost IFN-γ production; and (4) neural ablation techniques to disrupt immunosuppressive neural signals and promote cytotoxic T cell infiltration. These integrated strategies aim to overcome resistance mechanisms and optimize immune responses in gastric cancer^1^. This figure was created using Adobe Illustrator (version 27.9). ^1^α7nAChR: α7 nicotinic acetylcholine receptor; NK1: Neurokinin-1 receptor

Surgical and chemical denervation are being revisited for their capacity to reprogram the tumor-immune microenvironment in the cancer. In preclinical models, vagotomy or selective denervation is associated with reduced tumor growth, greater CD8^+^ T-cell infiltration, and lower Treg abundance, effects consistent with attenuation of cholinergic immunomodulatory inputs and decreased neurotrophic drive, such as nerve growth factor. Chemical denervation with botulinum toxin has yielded comparable signals in selected settings and is potentially reversible [107]. However, the timing and extent of denervation appear critical, as complete autonomic disruption can have unintended consequences on gastrointestinal function and may paradoxically activate alternative immunosuppressive pathways. Emerging techniques like focused ultrasound and radiofrequency ablation offer more targeted approaches to modulate specific nerve populations without complete disruption. These approaches are being explored in combination with immunotherapy, with early-phase clinical trials investigating whether localized nerve modulation can enhance checkpoint inhibitor efficacy while minimizing systemic side effects. The development of nerve-sparing techniques that selectively target tumor-associated nerves while preserving normal autonomic function represents an important direction for future research (Fig. 3). Specificity can be enhanced by nerve-sparing trajectories, receptor-pathway selection, and stage-aware timing that prioritizes tumor-proximal plexuses [108,109]. Safety remains central: extensive denervation may impair motility or bile reflux control and can induce dumping-like syndromes or collateral injury [110]; early studies should incorporate objective target-engagement and pharmacodynamic readouts (heart-rate variability, tissue/serum norepinephrine, tyrosine-hydroxylase–positive fiber density, pathway activation), standardized safety monitoring (orthostatic vitals, rhythm surveillance, gastric emptying), and predefined stopping rules [111]. These procedures are being prospectively combined with immune-checkpoint blockade to test whether localized neuromodulation improves efficacy while limiting systemic adverse effects, and nerve-sparing techniques that selectively address tumor-associated nerves represent a pragmatic direction for near-term clinical translation. Fig. 3 illustrates strategies that target the neuro-immune axis to enhance immunotherapy in gastric cancer.

## 5 Limitation and Future Perspectives

### 5.1 Current Limitations in Understanding the Neuro-Immune Axis in Gastric Cancer

The neuro-immune axis in gastric cancer is increasingly recognized as a critical determinant of tumor behavior and therapeutic response, yet its mechanisms remain incompletely defined. Most current evidence derives from reductionist preclinical models that only partially capture the dynamic interactions among neural, immune, stromal, and epithelial compartments in human disease [112–114].

Advanced technologies such as single-cell RNA sequencing and spatial transcriptomics are beginning to resolve neuro–immune niches and receptor–ligand topologies across tumor, myeloid, and lymphoid compartments, including β2-adrenergic, α7 nicotinic acetylcholine, neurokinin-1, and CALCRL pathways relevant to gastric cancer [115,116]. However, these approaches remain limited by sampling bias, batch effects, and the difficulty of integrating multi-omic datasets across patients and disease stages. Likewise, current functional models—such as patient-derived organoids and organoid–neuron co-cultures or assembloids—allow controlled perturbation of cholinergic, adrenergic, and neuropeptide signaling [117], but lack full vascularization, stromal complexity, and an intact adaptive immune compartment. These constraints limit causal inference and hinder precise delineation of the temporal dynamics of neuro-immune interactions during tumor initiation, progression, and treatment. *In vivo* models, including optogenetic or chemogenetic mouse systems with neural tract tracing [118], allow cell type–specific activation or ablation of vagal, sympathetic, and sensory circuits and have provided important mechanistic insights. Yet species differences, restricted observation windows, and difficulty modeling chronic human stress and comorbidities limit direct translation to patients. Collectively, these methodological constraints highlight that key aspects of the neuro-immune axis in gastric cancer—such as stage-dependent roles of neural circuits, the hierarchy among different neuropeptide pathways, and the integration of central and peripheral signals—remain insufficiently defined.

### 5.2 Translational and Clinical Challenges

Translating neuro-immune insights into clinical benefit faces several additional obstacles. First, there is a lack of validated biomarkers to identify patients most likely to benefit from neuromodulatory strategies. Although parameters such as perineural invasion burden, innervation density, tissue or serum norepinephrine concentrations, and expression of receptors including ADRB2, CHRNA7, TACR1, and CALCRL are promising, they have not been standardized or prospectively evaluated in gastric cancer cohorts. The temporal plasticity of neural and immune compartments during therapy further complicates biomarker development.

Second, the safety profile of sustained neuromodulation remains incompletely defined. Pharmacologic approaches such as β-blockers, NK1R antagonists, and agents targeting neurotrophic or neuropeptide pathways may exert off-tumor effects on the cardiovascular, autonomic, and central nervous systems. Device-based interventions, including vagus nerve stimulation and other bioelectronic strategies, likewise require careful evaluation of long-term impacts on homeostatic neural circuits. These considerations underscore the need for stepwise clinical development with rigorous safety monitoring and mechanistic endpoints, including PD-L1 dynamics, CD8^+^ effector function, dendritic-cell IL-12 production, myeloid-suppressor indices, and quantitative measures of innervation and neurotransmitter tone.

Third, evidence supporting the integration of traditional or complementary interventions into neuro-immune–focused treatment paradigms remains preliminary. Although compounds such as berberine [119], evodiamine [120], and ginsenosides [121] have been reported to modulate PD-L1 expression, TRPV1/Ca^2+^ coupling, or β2-adrenergic/CREB signaling, most available data derive from preclinical models with heterogeneous designs and limited mechanistic characterization in gastric cancer. Electroacupuncture [122] and related neuromodulatory procedures likewise appear capable of engaging defined vagal–adrenal and α7 nicotinic acetylcholine receptor–dependent pathways, but robust clinical evidence in gastric cancer is currently lacking. The absence of standardized intervention protocols, patient-selection criteria, and outcome measures remains a major barrier to translation.

### 5.3 Methodological Priorities and Future Experimental Platforms

Future progress will depend on next-generation experimental platforms designed to overcome these limitations. Immune-competent, patient-matched organoids incorporating autologous CD8^+^ T cells, NK cells, dendritic cells, and myeloid-derived suppressor cells, together with integrated enteric and sensory neurons derived from human induced pluripotent stem cells, could more faithfully recapitulate neuro-immune–tumor crosstalk. Axon-on-a-chip systems and microfluidic gut–brain platforms may enable controlled modeling of long-range neural circuits and microbiota-derived influences, whereas CRISPR-based perturb-seq approaches can generate causal maps linking receptors and pathways such as β2-AR, α7nAChR, NK1R, and CALCRL to defined immune and tumor phenotypes.

To ensure comparability across studies, we propose standardized functional readouts—including PD-L1 kinetics, dendritic-cell IL-12 production, CD8^+^ cytotoxicity, myeloid-derived suppressor cell indices, neurotransmitter levels, and neuropeptide signaling outputs—to benchmark neuro-immune interventions. Such platforms and metrics should sharpen mechanistic understanding and help prioritize the most promising targets for early-phase clinical trials.

### 5.4 Future Perspectives: toward Precision Neuro-Immune Modulation in Gastric Cancer

From a clinical standpoint, the growing recognition of neuro-immune heterogeneity in gastric cancer supports the development of more precise, individualized treatment strategies. Future classifications may incorporate neural infiltration patterns, neuropeptide expression profiles, autonomic signaling activity, and microbiota–gut–brain signatures alongside conventional histopathological and molecular markers. Patients with high adrenergic signaling or dense perineural invasion may be candidates for β-blockers or NK1R antagonists combined with standard chemotherapy or immune checkpoint inhibitors, whereas those with neuroinflammatory or vagal-dominant signatures could benefit from parasympathetic neuromodulation and agents that restore effective antitumor immunity.

The microbiota–gut–brain axis provides an additional layer of therapeutic opportunity. Rational modulation of the microbiome—using probiotics, microbiota-derived metabolites, or dietary interventions—may complement pharmacologic or device-based neuromodulation to reprogram the tumor microenvironment toward more robust immune control. Carefully designed combination trials that integrate neural-targeting strategies with cytotoxic, targeted, and immune therapies will be crucial to determine whether modulation of the neuro-immune axis can meaningfully overcome current therapeutic limitations in gastric cancer.

In summary, although substantial gaps remain in our understanding of the neuro-immune axis in gastric cancer and in the tools available to manipulate it safely and effectively, the field is moving toward more mechanistically informed and patient-tailored approaches. Addressing current limitations in experimental models, biomarker development, and clinical trial design—through coordinated efforts among basic scientists, clinicians, and bioengineers—will be essential to translate emerging insights into neural–tumor–immune crosstalk into tangible clinical benefit.

## 6 Conclusion

The neuroimmune axis is a key regulatory node for the progression and treatment of gastric cancer, and targeted intervention may improve the efficacy of immunotherapy.

## Data Availability

Not applicable. This article is a systematic review based on previously published studies; no new datasets were generated or analyzed.
